# Phonemic verbal fluency and age: A preliminary study

**DOI:** 10.1590/S1980-57642009DN20400017

**Published:** 2008

**Authors:** Veronique Agnes Guernet Steiner, Leticia Lessa Mansur, Sonia Maria D. Brucki, Ricardo Nitrini

**Affiliations:** 1 Rehabilitation Sciences Post Graduation Program. Medical School of São Paulo University.; 2 Department of Speech Pathology, Physiotherapy and Occupational Therapy. Medical School of São Paulo University.; 3 Research Group of Cognitive and Behavioral Neurology. Department of Neurology of the Medical School of São Paulo University.

**Keywords:** evaluation, ageing, verbal fluency, cognition

## Abstract

Verbal fluency (VF) has been one of the most frequently used instruments in clinics and research, both independently or included in a battery recommended for the detection of cognitive alterations. Phonemic-verbal fluency (PVF) is an interesting variant for cognitive diagnosis particularly because studies indicate that it is less influenced by age.

**Objectives:**

1. To estimate the effects of age on PVF tests in their original forms, with the /f/-/a/-/s/ phonemes. 2. To estimate the effects of the phoneme /p/ and compare it to the original form in item generation. 3. To verify associations between the Token Test (TT), Mini-Mental State Exam (MMSE) and depressive symptoms on performance with /f/-/a/-/s/-/p/ phonemes.

**Methods:**

Forty-eight healthy individuals with ages ranging from 30 to 80 years were evaluated with the MMSE, TT and PVF tests.

**Results:**

Age was correlated with the MMSE, TT and depressive symptoms. There was no association between age and performance on the fluency test, independent of the phoneme used. Among the socio-demographic factors studied, age had a significant impact on performance. There was no phoneme effect in item-generation, when comparing the traditional form of VF (/f/-/a/-/s/) and the /p/ phoneme.

**Conclusions:**

The traditional form of FAS is interchangeable with the modified presentation, therefore both forms may be used in clinical or research settings. PVF is a valuable approach for detecting cognitive alterations in the aged, given its stability throughout the ageing process.

## Abstract (pt)

A fluência verbal (FV) fonêmica tem sido um dos instrumentos mais frequentemente utilizados em atividades clínicas e de pesquisa, de modo isolado ou incluída em baterias recomendadas para a detecção de alterações cognitivas. A versão fonêmica da FV é uma variante interessante para o diagnóstico cognitivo, particularmente porque estudos indicam sua resistência aos efeitos do envelhecimento.

**Objetivos:**

1. Estimar os efeitos de envelhecimento na FV fonêmica, na forma original, com os fonemas /f/-/a/-/s/. 2. Estimar os efeitos de fonema/letra alternativo P e comparar com a forma original de geração de itens. 3. Verificar associações entre o Token Teste (TT), Mini-Exame do Estado Mental (MEEM) e sintomas depressivos na geração de itens com fonemas /f/-/a/-/s/-/p/.

**Métodos:**

Quarenta e oito indivíduos saudáveis com idade entre 30 e 80 anos foram avaliados com o MEEM, TT e testes de FV fonêmica.

**Resultados:**

A idade correlacionou-se com o MEEM, TT e sintomas depressivos. Não houve associação entre idade e desempenho no teste de fluência independentemente do fonema/letra utilizado. Tomando-se em consideração o conjunto de fatores sociodemográficos, a idade teve impacto significativo no desempenho. Não houve efeito de fonema na geração de itens, quando se comparou a forma tradicional, com F-A-S e o fonema P.

**Conclusões:**

A forma tradicional de apresentação do F-A-S pode ser intercambiada com a apresentação modificada, então é possível usar ambas as formas em atividade clínica e de pesquisa. A FV fonêmica é um instrumento interessante para detectar alterações cognitivas no idoso, dada sua estabilidade no processo de envelhecimento.

Thurstone^1^ conceived the verbal fluency (VF) test which entails retrieval of names of items beginning with the letters S and C, in written modality over a period of five minutes. Since then, the VF has remained one of the most-frequently used instruments both clinically and in research, independently or included in batteries recommended for the detection of cognitive alterations.^2^

The test has been exploited in numerous forms of presentation, varying in time and retrieval modality, restriction type and demand of executive control. One of the most-used versions requests the generation of words beginning with the phonemes /f/-/a/-/s/. This test is known as the Controlled Oral Word Association Test – COWAT. Other denominations are also used: word fluency, letter fluency, F-A-S test, phonemic fluency and controlled verbal fluency. Category fluency exploits various types of semantic criteria, such as names of animals, supermarket items, transportation means, among others.

The retrieval of items beginning with a specific phonemic criteria together with recuperation of semantic category, have been valuable clinical instruments for diagnoses, semiologic characterization, follow-up and prognoses^3^ in degenerative illnesses and depression.^4,5^

Traditionally, the number of timely acceptable answers is used as the principal variable in evaluating performance on VF tests. Some authors have proposed qualitative measures such as clustering (groupings or contiguous words in the same sub-category) and switching (changing groupings) to infer the nature of the deficit. Impaired clustering reflects compromise in the temporal regions of cortex, while switching is impaired in patients with compromised dorso-lateral and superior medial frontal^6^ regions, the latter being more associated to impaired working memory. A fall in switching performance is evident in advanced ages.^7,8^

The results from meta-analyses of studies on Alzheimer’s and Parkinson’s disease indicate that the phonemic criteria for item generation is more resistant to the effects of disease progression than semantics.^4,5^

Numerous normalizations of healthy populations with different ages, ethnicities and formal education have been carried out , most of them in the English language.^9,10^ There are also norms available for the Latin languages.^11^

The sets of results indicate a predominant effect of age for semantic-VF, and of formal education in phonemic-VF.^12-14^

A Brazilian study in the aged^15^ investigated both semantic and phonemic types of VF but no difference in phonemic and semantic criteria in the ability to distinguish AD, mild cognitive impairment and controls was observed.

The choice of the phonemes /f/-/a/-/s/ in the original version of the test is based upon the high frequency of these letters in English, a point which warrants attention when transposing these criteria for speakers of the Portuguese language. Senhorini^16^ indicated that /f/-/a/-/s/ were among the phonemes that generated a large number of items (14 were studied) by speakers of the Portuguese language, and found results comparable between English and Portuguese when the task was undertaken in its original format. This study did not include individuals that were older than 50 years.

The study of phonemic-VF in the aged population is especially interesting as this population is most susceptible to developing dementia. Given the task is little affected by the ageing process, it can be a useful instrument for the early diagnosis of degenerative diseases and other cognitive alterations.

It is possible that the number of items generated is influenced by the chosen phoneme. In Portuguese the phoneme /s/, for example, encompasses homophonic forms with /c/(before /e/, /i/) which can be a source of confusion. On the other hand, the phoneme /p/, considered easy in Portuguese, is one of the first introduced when learning to write and has been used in VF evaluations in clinical practice.

Bearing this in mind, the present study had the following objectives:

1. To estimate the effect of age on tests of phonemic fluency in their original format, using the phonemes /f/-/a/-s/.
2. To estimate the effects of the phoneme /p/ and compare these with the original format in item-generation.
3. To verify associations between the performance of the /f/-/a/-/s/-/p/ VF test and cognitive-behavioral measures.

## Methods

### Subjects

Forty-eight subjects, distributed uniformly between the ages of 30 to 80 years of age and of both genders (Table 1), were studied.

**Table 1:** Descriptive statistics of the sample (n=48)

	Min	Max	P25	P50	P75
Age	31	80	49	60	69
Years of education	11	15	13	15	15
Token Test	33	36	35	35	35
MMSE	26	30	27	29	30
Depression scale	3	6	3	4	5
F	5	26	12	15	19
A	8	25	12	15	18
S	4	28	13	15	18
Total /f/-/a/-/s/	22	74	38	46	56
P	7	33	14	17	21
Total /f/-/a/-/p/	26	79	39	48	56

The following inclusion criteria were used: greater than 10 years of formal education, absence of dementia according to the Brazilian version of the Mini-Mental State Exam (MMSE)^17^ and absence of depressive symptoms according to the Yesavage scale.^18^ The cut-off score for MMSE was 26 points^19^ and for Yesavage, greater than or equal to ten points.

For the purpose of selecting only healthy-ageing individuals, a questionnaire following the MOANS (Mayo Older American Normative Studies)^20^ criteria was devised. The objective of its application was to exclude subjects that were not living independently, reported having psychiatric or neurological diseases, presented complaints of cognitive difficulties, or were taking psychotropic medication that could compromise cognition or suggest neuropsychiatric disorders. To be included, the subjects had to answer negatively to all questions.

Subjects with speech, visual or hearing alterations with functional impact on communicative interaction where excluded.

Evaluation protocols were individually applied. Initially the subjects provided identification data and completed the MOANS and depression questionnaires. Subsequently, the evaluations were applied in the following order: the MMSE, Token Test – short version (TT)^21^ and VF tests.

In phonemic VF the subjects were asked to generate the greatest number of words beginning with /f/-/a/-/s/-/p/ for one minute respectively.

The study was approved by the Ethics Committee of Hospital das Clínicas of the Medical School of USP (CAPEPesq- process n. 0950/07) on February 11, 2008. All of the volunteers signed the Term of Free and Informed Consent.

The data were described and analyzed by Spearman’s Correlation Test and Multiple Linear Regression using the SPSS 15.0 Package.

## Results

Our sample was composed of 48 healthy control subjects whose characteristics are described in Table 1.

There was no association between years of schooling and VF measures, or between schooling and TT scores. Schooling correlated with age (r= –0.465; p<0.01); MMSE scores (r= –0.481; p<0.01) and depressive symptom scores (r= –0.347; p<0.05).

When age and its correlations were analyzed, significant associations with scores on TT (r= –0.542; p<0.01), MMSE (r= –0.480; p<0.01), and depressive symptoms (r=0.526; p<0.01) were observed. Age was found to have negative significant correlation with all fluency measures, although all of these were weak (Table 2).

**Table 2:** Results of Spearman's correlation test.

		Age	F	A	S	Total F-A-S	p
Age	r value						
F	r value	-0.325*					
A	r value	-0.387**	0.671**				
S	r value	-0.371**	0.610**	0.679**			
Total F-A-S	r value	-0.399**	0.876**	0.873**	0.862**		
P	r value	-0.292*	0.586**	0.578**	0.671**	0.705**	
Total F-A-P	r value	-0.395**	0.869**	0.852**	0.762**	0.951**	0.840**
	N	48	48	48	48	48	48

* Correlation is significant at the 0.05 level (2-tailed);

** Correlation is significant at the 0.01 level (2-tailed).

As expected, fluency measures had strong correlations with one another. There was very strong inter-correlation between total /f/-/a/-/s/ scores and total /f/-/a/-/p/ scores, a factor allowing interchangeability between these letters in clinical evaluation. No correlation among TT scores and verbal fluency measures was observed (total of /f/-/a/-/s/, total /f/-/a/-/p/, or by any single phoneme).

In Multiple Linear Regression, introducing age and schooling as predictors, only age was found to be significant for performance on phonemic fluency measures in this sample (Table 3).

**Table 3:** Results of multiple linear regression test.

	F value	sig	Beta coefficient	T	sig
Total F-A-S	4.701	0.014			
Predictors					
Age			-0.418	-2.773	0.008
Schooling			-0.006	-0.038	0.969
Total F-A-P	4.353	0.019			
Predictors					
Age			-0.416	-2.739	0.009
Schooling			-0.032	-0.212	0.833

We noted that independent of phonemes used, the results remained the same. However, age influenced the results in this sample of highly educated subjects.

## Discussion

Although this sample included individuals with greater than ten-years of schooling we noted that VF was influenced by access to a higher than average level of education.

The discrete association between aging and schooling revealed a profile of the Brazilian aged population in which older individuals have less formal education.

The correlation between age and the TT was somewhat expected, given this instrument demands working-memory resources, sensitive to the aging process. The negative effect of increased age and performance on the MMSE, was indicated in previous studies^22^ and has been confirmed in studies on the Brazilian population.^23^ Although age-associated depressive-symptoms were not significant, they cannot be ignored since they may constitute an early sign of cognitive disease^24^ and have an impact on functioning in daily-life.^25^

At first glance, the absence of correlation between the TT and phonemic VF calls attention, because the two instruments are associated with working-memory demands. The TT requires the temporary storage and manipulation of data in situations of auditory comprehension, while the phonemic VF requires temporary manipulation in an active situation of generation, which constitutes an important differential in identifying manipulation strategies employed.

The correlation of age with all other phonemes was weak and not expressed significantly in comparison to the means of young, adult, young elderly and older elderly age groups. Our study showed that the discrete effect of age did not interact with the effect of the phonemes even in the older populations. The different phonemes evaluated did not indicate differences in the number of items generated. This was the case both in the conventional presentation of /f/-/a/-/s/ and the version using the phoneme /p/. It is worth noting that the sample studied had a high level of formal education, which could account for similar performance independently of the phoneme used. Moreover, the phonemic VF test is more sensitive to the effect of schooling than the semantic VF. This indicates the need to evaluate populations with a lesser degree of schooling in order to highlight the impact of formal instruction on these results.

The weight of the age variable among other socio-demographic factors highlights the effect of this factor on our sample, controlled for schooling and cognitive aspects.

This preliminary finding was consistent with previous studies which indicated maintenance of skills to generate items by phonemic criteria even in more advanced ages in highly educated populations, thereby suggesting the use of this type of evaluation to diagnose cognitive alterations frequent in aging.

Although qualitative analysis of clusters and switching was not carried out, we know that the PVF test induces associations where there are a smaller number of clusters and lesser control of switches, compared to the semantic-VF. This hypothesis was not confirmed in studies^7^ where age contributed to the number of switches produced and to the total number of items generated. It is possible that our aged employed this control, given that they were efficient and the number of items generated was similar to the other age groups.

Maintaining the test in its original format is advantageous because it facilitates data comparison on an international scale. Such comparisons, however, would require population samples with a greater number of participants.

### Conclusions

1. The traditional form of /f/-/a/-/s/ is interchangeable with the modified presentation.
2. It is possible to use both forms in clinical or research settings.
3. Phonemic verbal fluency is an valuable approach for detecting cognitive alterations in the aged, given its stability throughout the ageing process.

## References

[1] Thurstone LL (1938). Primary Mental Abilities.

[2] Nitrini R, Caramelli P, Bottino CMC, Damasceno BP, Brucki SMD, Anghinah R (2005). Diagnóstico de doença de Alzheimer no
Brasil. Arq Neuropsiquiatr.

[3] Cosentino S, Scarmeas N, Albert SM, Stern Y (2006). Verbal fluency predicts mortality in Alzheimer
disease. Cogn Behav Neurol.

[4] Henry JD, Crawford JR (2004). Verbal fluency deficits in Parkinson's disease: a
meta-analysis. J Int Neuropsychol Soc.

[5] Henry JD, Crawford, Phillips LH (2004). Verbal fluency performance in dementia of the Alzheimer's type: a
meta-analysis. Neuropsychologia.

[6] Troyer AK, Moscovitch M, Winocur G, Alexander MP, Stuss D (1998). Clustering and switching on verbal fluency: the effects of focal
temporal and temporal-lobe lesions. Neuropsychologia.

[7] Kosmidis MH, Vlahou CH, Panagiotaki P, Kiosseoglou G (2004). The verbal fluency task on the Greek population: normative data
and clustering and switching strategies. J Int Neuropsychol Soc.

[8] Troyer AK, Moscovitch M, Winocur G (1997). Clustering and switching as two components of verbal fluency:
evidence from younger and healthy adults. Neuropsychology.

[9] Steinberg BA, Bieliauskas LA, Smith GE, Ivnik RJ (2005). Mayo's Older Americans Normative Studies: Age and IQ-adjusted
norms for the Trail-Making Test, the Stroop Test and MAE Controlled Oral
Word Association Test. Clin Neuropsychol.

[10] Lucas JA, Ivnik RJ, Smith GE (2005). Mayo's Older African Americans Normative Studies: norms for
Boston Naming Test, Controlled Oral Word Association, Category Fluency,
Animal Naming, Token Test, WRAT Reading, Trail Making Test, Stroop Test and
Judgement of Line Orientation. Clin Neuropsychol.

[11] Gonzalez HM, Mungas D, Haan MN (2005). A semantic verbal fluency for English and Spanish-speaking older
Mexican-americans. Arch Clin Neuropsychol.

[12] Tombaugh TN, Kozak J, Rees L (1999). Normative data stratified by age and education for two measures
of verbal fluency: FAS and animal naming. Arch Clin Neuropsychol.

[13] Loonstra AS, Tarlow AR, Sellers AH (2001). COWAT metanorms across age, education, and gender. Appl Neuropsychol.

[14] Tun PA, Lachman ME (2006). Telephone assessment of cognitive function in adulthood: the
Brief Test of Adult Cognition by Telephone. Age Ageing.

[15] Hamdan AC, Bueno OFA (2005). Relações entre controle executivo e memória
episódica verbal no comprometimento cognitivo leve e na
demência tipo Alzheimer. Estud Psicol(Natal).

[16] Senhorini MC, Amaro Jr E, de Mello Ayres A, de Simone A, Busatto GF (2006). Phonemic fluency in Portuguese-speaking subjects in Brazil:
ranking of letters. J Clin Exp Neuropsychol.

[17] Folstein M, Folstein S, Mc Hugh P (1975). Mini-mental state: A practical method for grading the cognitive
state of patients for the clinician. J Psychiatr Res.

[18] Yesavage JA, Brink TL, Rose TL, Lum O (1983). Development and validation of a geriatric depression scale: a
preliminary report. J Psychat Res.

[19] Brucki SM, Nitrini R, Caramelli P, Bertolucci PH, Okamoto IH (2003). Suggestions for utilization of the mini-mental state examination
in Brazil. Arq Neuropsiquiatr.

[20] Smith GE, Ivnik RJ, Petersen RC (2003). Normative neuropsychology. Mild Cognitive Impairment.

[21] De Renzi E, Faglioni P (1978). Normative data and screening power of a shortened version of the
Token Test. Cortex.

[22] Doufuil C, Clayton D, Brayne C (2000). Population norms for the MMSE in the very old: Estimates based on
longitudinal data. Neurology.

[23] Castro-Costa E, Fuzikawa C, Uchoa E, Firmo JOA, Lima-Costa MF (2008). Norms for the Mini-Mental State Examination. Arq Neuropsiquiatr.

[24] Rosenberg PB, Lyketos CF (2008). Mild cognitive impairment: searching for the prodrome of
Alzheimer's disease. World Psychiatry.

[25] Santos JLF, Lebrão ML, Duarte YAO, Lima FD (2003). Functional performance of the elderly in instrumental activities
of daily living: an analysis in the municipality of São Paulo,
Brazil. Cad Saúde Pública.

